# Racial Disparities in Receipt of Guideline-Concordant Care in Older Adults With Early Breast Cancer

**DOI:** 10.1001/jamanetworkopen.2024.41056

**Published:** 2024-10-24

**Authors:** Brenda S. Castillo, Taussia Boadi, Xiaoyan Han, Lawrence N. Shulman, Yehoda M. Martei

**Affiliations:** 1 Department of Medicine, Perelman School of Medicine, University of Pennsylvania, Philadelphia; 2 University of Pennsylvania, Philadelphia; 3 Perelman School of Medicine, University of Pennsylvania, Philadelphia; 4 Hematology-Oncology Division, Department of Medicine, University of Pennsylvania, Philadelphia; 5 Abramson Cancer Center, University of Pennsylvania, Philadelphia

## Abstract

**Importance:**

Racial disparities in receipt of guideline-concordant care (GCC) among older patients with potentially curable breast cancer are understudied.

**Objective:**

To determine whether rates of GCC, time to treatment initiation, and all-cause mortality in stage I to III breast cancer differ by race among older adults.

**Design, Setting, and Participants:**

This cohort study used data from the National Cancer Database and included patients aged 65 years and older with stage I to III breast cancer, diagnosed between 2010 and 2019. Data analysis was conducted between July 2022 to July 2023.

**Exposures:**

Race, defined as non-Hispanic Black or non-Hispanic White.

**Main Outcomes and Measures:**

The primary outcome was nonreceipt of GCC, defined using the National Comprehensive Cancer Network guidelines, and all-cause mortality. The secondary outcome was time to treatment initiation. Univariate and multivariate regression analysis were used to determine association between exposure and outcomes. Models for GCC and all-cause mortality included age, stage, receptor status, year of diagnosis, Charlson-Deyo comorbidity index, insurance, health care setting, and neighborhood-level educational attainment and median income.

**Results:**

The analytic cohort included 258 531 participants (mean [SD] age, 72.5 [6.0] years), with 25 174 participants who identified as non-Hispanic Black (9.7%) and 233 357 participants who identified as non-Hispanic White (90.3%), diagnosed between 2010 and 2017. A total of 4563 non-Hispanic Black participants (18.1%) and 35 374 non-Hispanic White participants (15.2%) did not receive GCC. Non-Hispanic Black race, compared with non-Hispanic White race, was associated with increased odds of not receiving GCC in the multivariate analysis (adjusted odds ratio [aOR], 1.13; 95% CI, 1.08-1.17; *P* < .001). Non-Hispanic Black race was associated with 26.1% increased risk of all-cause mortality in the univariate analysis, which decreased to 4.7%, after adjusting for GCC and clinical and sociodemographic factors (adjusted hazard ratio, 1.05; 95% CI, 1.01-1.08; *P* = .006). Non-Hispanic White race, compared with non-Hispanic Black race, was associated with increased odds of initiating treatment within 30 (OR, 1.65; 95% CI, 1.6-1.69), 60 (OR, 2.11; 95% CI, 2.04-2.18), and 90 (OR, 2.39; 95% CI, 2.27-2.51) days of diagnosis.

**Conclusions and Relevance:**

In this cohort study, non-Hispanic Black race was associated with increased odds of not receiving GCC and less timely treatment initiation. Non-Hispanic Black race was associated with increased all-cause mortality, which was reduced after adjusting for GCC and clinical and sociodemographic factors. These findings suggest that optimizing timely receipt of GCC may represent a modifiable pathway to improving inferior survival outcomes among older non-Hispanic Black patients with breast cancer.

## Key Points

**Question:**

Are there racial disparities in receipt of guideline-concordant care (GCC) and all-cause mortality among patients aged 65 years or older with stage I-III breast cancer?

**Findings:**

In this cohort study including 258 531 patients, non-Hispanic Black race, compared with non-Hispanic White race, was associated with a lower likelihood of receiving GCC and increased risk of all-cause mortality, which decreased in the adjusted model including receipt of GCC and clinical and sociodemographic factors. Non-Hispanic Black race was associated with reduced odds of timely treatment initiation.

**Meaning:**

These findings suggest that optimizing timely access and receipt of GCC in older adults may represent a modifiable pathway for mitigating racial differences in all-cause mortality among patients with early breast cancer.

## Teaser

This cohort study examines the association of non-Hispanic Black or non-Hispanic White race with receipt of guideline-concordant care among older adults with stage I to III breast cancer.

## Introduction

Breast cancer mortality has declined significantly over the last 4 decades, which is partly attributable to improved systemic treatment for early-stage breast cancer.^1^ However, disparities in breast cancer survival have been reported in different demographic groups, with disproportionately worse breast cancer–specific and overall survival outcomes among older patients and individuals who identify as non-Hispanic Black following a diagnosis of breast cancer.^2,3,4^ Although individuals aged 70 years and older represent one-third of patients with incident breast cancer annually, 47% of all breast cancer–specific deaths occur in this demographic group.^5^ Similarly, non-Hispanic Black race is associated with an age-adjusted breast cancer mortality rate that is 40% higher than among non-Hispanic White individuals with breast cancer.^6,7,8^

The reasons for the survival disparities in these demographic groups are complex and multifactorial but may be partly associated with disparities in receipt of guideline-concordant cancer care and timeliness of therapy initiation along the cancer care continuum.^9,10,11^ Among patients with breast cancer aged 65 years and older, omission of guideline-indicated multimodality treatment, including adjuvant radiation for breast conservation surgery, systemic chemotherapy, and targeted therapy, have been documented.^12,13,14,15^ Risk factors associated with undertreatment in older adults include competing comorbidities, frailty, life expectancy, and functional status.^16^ Additionally, it is argued that older adults are grossly underrepresented in therapeutic cancer clinical trials, limiting external generalizability, particularly regarding the tolerability of systemic therapy in older adults with preexisting comorbidities.^17^ However, an assessment of these risks and benefits suggests that undertreatment may lead to preventable deaths in older adults.^18^

Although non-Hispanic Black race is associated with a disproportionately higher rate of triple-negative breast cancer, recent studies have also shown that barriers to access may be more significant modifiable contributors to the observed racial disparities in survival.^2,19^ Non-Hispanic Black race has also been associated with nonreceipt of all forms of curative treatment for breast cancer, including definitive surgery, reconstructive surgery, systemic treatment, and radiation therapy, when indicated.^20,21,22^

Although there is robust literature on disparities in these 2 populations, few studies have examined guideline-concordant therapy receipt and survival disparities for older non-Hispanic Black adults who are at the intersection of these 2 marginalized populations. Our objectives were to evaluate receipt of guideline-concordant care (GCC) for non-Hispanic Black compared with non-Hispanic White older adults with stage I to III breast cancer and evaluate all-cause mortality for non-Hispanic Black compared with non-Hispanic White older adults with stage I to III breast cancer, adjusting for receipt of GCC. In secondary analysis, we evaluated racial differences in time to treatment initiation (TTI).

## Methods

This cohort study was assessed by the University of Pennsylvania institutional review board on February 23, 2022, as not meeting requirement for human participants research or informed consent per 45 CRF §46.102(f). This study is reported following the Strengthening the Reporting of Observational Studies in Epidemiology (STROBE) reporting guideline.

### Study Design and Cohort Selection

**Figure 1.**
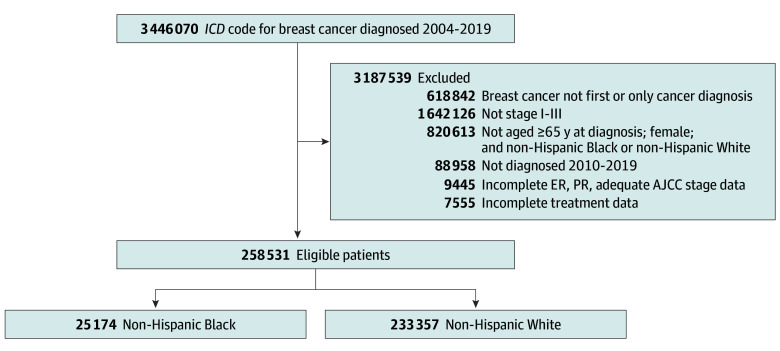
Cohort Selection Flowchart AJCC indicates American Joint Committee on Cancer; ER, estrogen receptor; *ICD*, *International Classification of Diseases, Ninth Revision* or *International Statistical Classification of Diseases and Related Health Problems, Tenth Revision* (depending on year of diagnosis); PR, progesterone receptor.

We conducted a retrospective cohort analysis of patients with breast cancer in the National Cancer Database (NCDB), a joint program of the Commission on Cancer of the American College of Surgeons and the American Cancer Society, which is a nationwide oncology outcomes database that captures approximately 70% of all newly diagnosed cancers in the US.^23^ Data requisition was completed in June 2022, and the analysis was conducted from July 2022 to June 2023. The analytic cohort included female patients aged at least 65 years and diagnosed with stage I to III breast cancer as their first and only cancer diagnosed between 2010 and 2019 (Figure 1). We also included only patients who were identified as non-Hispanic Black or non-Hispanic White race in the NCDB. Race were designated as self-reported information in the cancer registries comprising the NCDB. The exclusion criteria were stage 0 (in situ–only disease), M1 metastatic disease, or stage IV disease. Additionally, we excluded individuals with missing stage, receptor status, surgery, systemic treatment, and radiation therapy data. Subsequently, patients who died prior to initiating any treatment were excluded from our analytic cohort.

### Measures and Definitions

**Figure 2.**
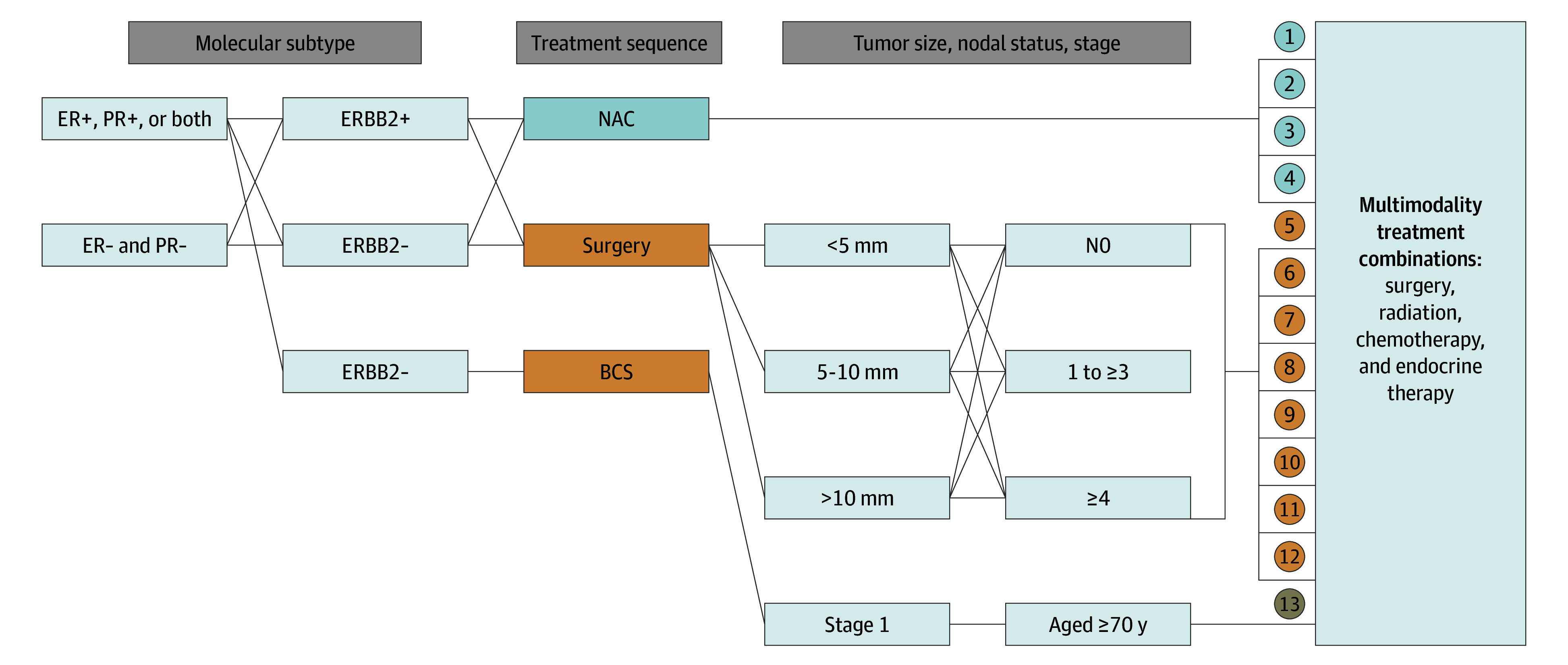
Guideline Concordant Care Algorithm BCS indicates breast conservation surgery; ER, estrogen receptor; ERBB2, human epidermal growth factor receptor 2 (formerly HER2); PR, progesterone receptor; −, negative; + positive.

We operationalized GCC as multimodality treatment, including surgery, radiation therapy and systemic treatment, as a binary outcome. The National Comprehensive Cancer Network Guidelines were used to define GCC. The respective guideline revisions between 2010 and 2019 were considered to account for trends and changes in treatment recommendations. The algorithm for GCC was developed using receptor status (estrogen receptor [ER], progesterone receptor [PR], human epidermal growth factor receptor 2 [ERBB2, formerly HER2]), stage, tumor size, nodal status, age, sequencing of systemic therapy (neoadjuvant vs adjuvant), systemic therapy choice, surgery, and receipt of radiation therapy (Figure 2). We identified 13 core combinations that were further subdivided into 43 possible pathways used to define GCC. Patients whose treatment pathways were consistent with any of the 43 pathways were defined as receiving GCC. Patients whose treatment pathway deviated from all 43 were assessed as not receiving GCC.

Of note, the NCDB database did not include specific systemic therapies; therefore, detailed systemic GCC could not be assessed. Information on genomic testing was not captured in the database; therefore, patients who would have met clinical criteria for genomic testing were assessed as meeting criteria for GCC whether or not they received additional chemotherapy in addition to endocrine therapy. This is a conservative approach in our analysis that is likely to bias our results toward the null by overestimating the number of patients who meet criteria for GCC. Lastly, consistent with current guidelines, omission of adjuvant radiation therapy in patients with low risk, those aged 70 years or older, and those with hormone-positive disease following breast-conserving surgery was considered GCC.^24,25^

We conducted Kaplan-Meier survival analysis to estimate survival from time of diagnosis to time of death (event). Patients were followed from diagnosis until the occurrence of death and were censored if they were lost to follow-up or were alive at last contact. The NCDB does not contain cause of death. Follow-up time was captured in the NCDB as number of months from diagnosis to event or censoring. Kaplan-Meier survival curves were generated stratified by non-Hispanic Black and non-Hispanic White race.

In secondary analysis, we assessed the proportions of non-Hispanic White compared with non-Hispanic Black patients who initiated cancer-directed treatment within 30 days, 60 days, and 90 days of diagnosis. These intervals are representative of the variations in benchmarks for cancer treatment initiation. Depending on whether the patient received neoadjuvant or adjuvant systemic therapy, TTI was assessed as number of days from breast cancer diagnosis to systemic therapy or surgery, respectively.

Independent covariates (and rationale for inclusion) were age (65-74 or ≥75 years, because prior studies have shown that GCC decreases significantly for patients aged ≥75 years), clinical or pathologic stage at diagnosis (I-III), receptor status (ER- and/or PR- positive and ERBB2-negative, triple-negative breast cancer, or ERBB2-positive, considered clinically meaningful tumor characteristics for treatment response and prognosis), year of diagnosis (to capture variations over time), and Charlson-Deyo comorbidity index, which was captured in NCDB as a weighted score derived from the sum of scores for different comorbid conditions and designated as 0 to 3, with 0 indicating no comorbidities. Patient demographics, including insurance status at the time of diagnosis, health care setting (urban vs rural were designated based on metropolitan or urban classifications vs rural, as specified in the NCDB), and neighborhood-level educational attainment (<14% or ≥14% with no high school degree) and income level (<$35 000 vs ≥$35 000 per year) were precoded in the data requisition file by matching the zip code of the patient at the time of diagnosis against the respective American Community Survey data. Raw zip code data were not included in the data requisition.

### Statistical Analysis

We summarized the distribution of patient, clinicopathologic, and demographic characteristics stratified by race. We reported counts and proportions for the categorical variables. We evaluated the association of non-Hispanic Black compared with non-Hispanic White race with lack of receipt of GCC using univariate and multivariate logistic regression, adjusting for clinical and sociodemographic variables. We estimated the hazard ratio (HR) with corresponding 95% CI for all-cause mortality in non-Hispanic Black patients compared with non-Hispanic White patients using Cox proportional hazards multivariate regression analysis, adjusting for GCC as a time-dependent covariate. Both the multivariable logistic regression model and the Cox proportional hazards model were adjusted for race, age, stage, year of diagnosis, receptor status, health care setting, insurance status, total Charlson-Deyo comorbidity index score, neighborhood-level median income and high school education attainment, and residence in urban vs rural county. eFigure 1 in Supplement 1 shows the conceptual model for this analysis. We used univariate analysis to estimate the odds ratio (OR) of treatment receipt within 30, 60, and 90 days from time of diagnosis in non-Hispanic White patients compared with non-Hispanic Black patients. All tests were conducted using 2-sided *P* < .05 to conclude statistical significance. Analyses were conducted using SAS software version 9.4 (SAS Institute).

## Results

The analytic cohort included 258 531 eligible patients (mean [SD] age, 72.5 [6.0] years), with 25 174 non-Hispanic Black patients (9.7%) and 233 357 non-Hispanic White patients (90.3%) diagnosed between 2010 and 2017. A total of 7555 patients with incomplete treatment data were excluded (eTable 1 in Supplement 1). Nine patients were listed as diagnosed in 2018 and there were no patients diagnosed in 2019 who met eligibility criteria; therefore, the regression analysis was limited to patients diagnosed between 2010 and 2017. The detailed distribution of baseline sociodemographic and clinical characteristics assessed are summarized and grouped by race in Table 1.

**Table 1.** Baseline Participant Demographics and Clinical Characteristics Grouped by Race

Characteristic	Patients, No. (%)		
	All (N = 258 531)	Non-Hispanic White (n = 233 357)	Non-Hispanic Black (n = 25 174)
Disease stage			
I	183 767 (71.1)	167 842 (71.9)	15 925 (63.3)
II	63 770 (24.7)	56 111 (24.0)	7659 (30.4)
III	10 994 (4.3)	9404 (4.0)	1590 (6.3)
Age, y			
Mean (SD)	72.5 (6.0)	72.5 (6.0)	72.0 (5.9)
Median (IQR)	71.0 (68.0-76.0)	71.0 (68.0-76.0)	71.0 (67.0-76.0)
65 to <75	175 106 (67.7)	157 419 (67.5)	17 687 (70.3)
≥75	83 425 (32.3)	75 938 (32.5)	7487 (29.7)
ER Status			
Positive	228 274 (88.3)	208 466 (89.3)	19 808 (78.7)
Negative	29 434 (11.4)	24 175 (10.4)	5259 (20.9)
Unknown/missing	823 (0.3)	716 (0.3)	107 (0.4)
PR status			
Positive	201 641 (78.0)	184 663 (79.1)	16 978 (67.4)
Negative	55 764 (21.6)	47 701 (20.4)	8063 (32.0)
Unknown/missing	1126 (0.4)	993 (0.4)	133 (0.5)
ERBB2 Status			
Positive	20 359 (7.9)	18 089 (7.8)	2270 (9.0)
Negative	236 669 (91.5)	213 988 (91.7)	22 681 (90.1)
Unknown/missing	1503 (0.6)	1280 (0.5)	223 (0.9)
Year of diagnosis			
2010	24 065 (9.3)	21 782 (9.3)	2283 (9.1)
2011	27 732 (10.7)	25 164 (10.8)	2568 (10.2)
2012	30 127 (11.7)	27 303 (11.7)	2824 (11.2)
2013	32 357 (12.5)	29 207 (12.5)	3150 (12.5)
2014	33 705 (13.0)	30 508 (13.1)	3197 (12.7)
2015	35 501 (13.7)	32 055 (13.7)	3446 (13.7)
2016	37 011 (14.3)	33 210 (14.2)	3801 (15.1)
2017	38 033 (14.7)	34 128 (14.6)	3905 (15.5)
Insurance payer			
Not insured	773 (0.3)	602 (0.3)	171 (0.7)
Private insurance or managed care	36 412 (14.1)	32 387 (13.9)	4025 (16.0)
Medicaid	2860 (1.1)	2103 (0.9)	757 (3.0)
Medicare	214 902 (83.1)	195 076 (83.6)	19 826 (78.8)
Other government	1106 (0.4)	1012 (0.4)	94 (0.4)
Unknown	2478 (1.0)	2177 (0.9)	301 (1.2)
Neighbor-level population with no high school degree, %			
Missing/unknown	33 609 (13.0)	30 194 (12.9)	3415 (13.6)
<14	96 219 (37.2)	92 113 (39.5)	4106 (16.3)
≥14	128 703 (49.8)	111 050 (47.6)	17 653 (70.1)
Neighborhood-level median income, $			
Missing/unknown	33 469 (12.9)	30 016 (12.9)	3453 (13.7)
<35 000	59 271 (22.9)	48 614 (20.8)	10 657 (42.3)
≥35 000	165 791 (64.1)	154 727 (66.3)	11 064 (44.0)
Facility type			
Community	136 477 (52.8)	125 934 (54.0)	10 543 (41.9)
Academic/research program	65 898 (25.5)	56 764 (24.3)	9134 (36.3)
Integrated Network Cancer Program	56 156 (21.7)	50 659 (21.7)	5497 (21.8)
Urban or rural setting			
Missing/unknown	5447 (2.1)	5095 (2.2)	352 (1.4)
Metropolitan areas or urban	247 782 (95.8)	223 232 (95.7)	24 550 (97.5)
Completely rural	5302 (2.1)	5030 (2.2)	272 (1.1)
Charlson-Deyo comorbidity index score			
0	200 417 (77.5)	183 409 (78.6)	17 008 (67.6)
1	43 762 (16.9)	37 811 (16.2)	5951 (23.6)
2	10 238 (4.0)	8743 (3.7)	1495 (5.9)
≥3	4114 (1.6)	3394 (1.5)	720 (2.9)
Receptor status			
Unknown	1549 (0.6)	1320 (0.6)	229 (0.9)
ER-positive or PR-positive and ERBB2-negative	216 748 (83.8)	197 973 (84.8)	18 775 (74.6)
ERBB2-positive	20 359 (7.9)	18 089 (7.8)	2270 (9.0)
ER-negative, PR-negative, and ERBB- negative	19875 (7.7)	15975 (6.8)	3900 (15.5)

Abbreviations: ER, estrogen receptor; ERBB2, human epidermal growth factor receptor 2 (formerly HER2); PR, progesterone receptor.

### GCC

Overall, 39 937 of 258 531 patients (15.4%) did not receive guideline-concordant multimodality therapy. Of 39937 patients who did not receive GCC, 4563 patients were non-Hispanic Black (11.4% ) and 35 374 patients were non-Hispanic White (88.6%). Within racial groups, 18.1% of non-Hispanic Black patients did not receive GCC, compared with 15.2% of non-Hispanic White patients. In the univariate analysis, non-Hispanic Black race was associated with increased odds of not receiving GCC (OR, 1.24; 95% CI, 1.20-1.28). In the adjusted model with any given level of clinical and demographic variables, non-Hispanic Black race was associated with increased odds of nonreceipt of GCC (adjusted OR [aOR], 1.13; 95% CI, 1.08-1.17). All the considered covariates in the adjusted model, except high school attainment, were significantly associated with not receiving GCC, representing their direct association with GCC not mediated by race (Table 2).

**Table 2.** Univariate and Multivariate Logistic Regression Analysis of Factors Associated With Nonreceipt of Guideline-Concordant Care Among Older Adults With Early Breast Cancer

Characteristic	Univariate analysis		Multivariate analysis	
	OR (95% CI)	*P* value	OR (95% CI)^a^	*P* value
Age, y				
65-74	1 [Reference]	<.001	1 [Reference]	<.001
≥75	2.24 (2.19-2.29)		2.29 (2.24-2.35)	
Race				
Non-Hispanic Black	1.24 (1.20-1.28)	<.001	1.13 (1.08-1.17)	<.001
Non-Hispanic White	1 [Reference]		1 [Reference]	
Disease stage				
I	1 [Reference]	<.001	1 [Reference]	<.001
II	1.97 (1.92-1.01)		1.78 (1.73- 1.82)	
III	3.53 (3.38-3.67)		3.04 (2.89-3.19)	
Year				
2010	1 [Reference]	<.001	1 [Reference]	<.001
2011	0.87 (0.83-0.91)		0.93 (0.88-0.98)	
2012	0.83 (0.87-0.91)		0.97 (0.92-1.02)	
2013	0.89 (0.85-0.93)		1.07 (1.01-1.12)	
2014	0.95(0.85-0.93)		1.08 (1.03-1.14)	
2015	0.90 (0.86-0.94)		1.12 (1.07-1.18)	
2016	0.85 (0.81-0.88)		1.07 (1.01-1.12)	
2017	0.91 (0.87-0.95)		1.19 (1.14-1.26)	
Receptor status				
ER-, PR-, and ERBB2-negative	1 [Reference]	<.001	1 [Reference]	<.001
ER-positive and/or PR-positive and ERBB2-negative	0.95 (0.91-0.99)		1.03 (0.98-1.08)	
ERBB2-positive	3.97 (3.78-4.17)		4.03 (3.82-4.26)	
Facility type				
Community	1 [Reference]	.009	1 [Reference]	<.001
Academic or research program	1.03 (1.00-1.05)		1.08 (1.05-1.12)	
Integrated Cancer Network Program	0.98 (0.95-1.00)		1.02 (0.98-1.05)	
Insurance status				
Medicaid, Medicare, or other government	1 [Reference]	<.001	1 [Reference]	<.001
Private or managed care	0.88 (0.85-0.90)		0.98 (0.94-1.02)	
Uninsured	1.43 (1.31-1.56)		1.27 (1.15-1.41)	
Charleson-Deyo comorbidity index score				
0	1 [Reference]	<.001	1 [Reference]	<.001
1	1.13 (1.1-1.17)		1.09 (1.05-1.12)	
2	1.48 (1.41-1.55)		1.39 (1.32-1.47)	
≥3	1.72 (1.6-1.85)		1.56 (1.43-1.7)	
Neighborhood-level population without high school degree, %				
<14	1 [Reference]	<.001	1 [Reference]	.14
≥14	1.11 (1.09-1.13)		1.02 (0.99-1.05)	
Neighborhood-level media income, $		<.001		.006
<35 000	1 [Reference]		1 [Reference]	
≥35 000	0.90 (0.88-0.93)		0.97 (0.94-1.00)	
Setting		.03		.008
Metropolitan areas or urban	1 [Reference]		1 [Reference]	
Rural	0.92 (0.85-0.99)		0.89 (0.81-0.97)	

Abbreviations: ER, estrogen receptor; ERBB2, human epidermal growth factor receptor 2 (formerly HER2); OR, odds ratio; PR, progesterone receptor.

^a^ The multivariable logistic regression model estimates the association of race with nonreceipt of guideline concordant care, adjusting for age, stage, year of diagnosis, receptor status, facility type, insurance, Charleston-Deyo comorbidity index, educational attainment, income, and setting.

### Overall Survival Analysis

A total of 5221 patients who identified as non-Hispanic Black (20.7%) and 40 557 patients who identified as non-Hispanic White (17.4%) were reported as dead at last contact. The median (IQR) follow-up time for the entire cohort was 63.4 (44.7-87.3) months. The Kaplan-Meier Survival analysis and life tables showing a breakdown of number at risk are shown in eFigure 2 in Supplement 1. In the univariate analysis, non-Hispanic Black race was significantly associated with an increased risk of all-cause mortality (HR, 1.26; 95% CI, 1.23-1.30; *P* < .001); in the adjusted model including time-dependent GCC as a mediator of mortality, the association of non-Hispanic Black race with all-cause mortality was reduced but remained significant (adjusted HR [aHR], 1.05; 95% CI, 1.01-1.08; *P* = .006). Furthermore, in the adjusted model, receiving GCC was associated with a 19% reduction in all-cause mortality (aHR, 0.81; 95% CI, 0.77-0.85; *P* < .001) at all levels of covariates. Lastly, neighborhood-level lower educational attainment and lower median income were also independently associated with a decreased risk of mortality. The largest magnitude of association for all-cause mortality not mediated by GCC were being aged 75 years or older, having stage III disease, and having a higher Charlson-Deyo comorbidity index score. All the covariates included in the model in the univariate and multivariate Cox regression analysis are summarized in Table 3.

**Table 3.** Multivariate Cox Regression Analysis of Factors Associated With All-Cause Mortality Among Older Adults With Early Breast Cancer

Characteristic	Univariate analysis		Multivariate analysis	
	HR (95% CI)	*P* value	HR (95% CI)	*P* value
Age, y				
65-74	1 [Reference]	<.001	1 [Reference]	<.001
≥75	2.61 (2.56-2.66)		2.50 (2.45-2.55)	
Race				
Non-Hispanic Black	1.26 (1.23-1.30)	<.001	1.05 (1.01-1.08)	.006
Non-Hispanic White	1 [Reference]		1 [Reference]	
Disease stage				
I	1 [Reference]	<.001	1 [Reference]	<.001
II	2.07 (2.03-2.11)		1.85 (1.81-1.89)	
III	3.91 (3.79-4.03)		3.39 (3.28-3.51)	
Diagnosis year				
2010	1 [Reference]	<.001	1 [Reference]	.005
2011	1.00 (0.97-1.03)		1.02 (0.98-1.05)	
2012	0.99 (0.96-1.02)		1.02 (0.96-1.06)	
2013	0.96 (0.93-0.99)		0.99 (0.96-1.04)	
2014	0.95 (0.92-0.99)		1.01 (0.97-1.05)	
2015	0.93 (0.90-0.97)		0.99 (0.96-1.04)	
2016	0.91 (0.87-0.95)		0.07 (0.92-1.01)	
2017	0.87 (0.83-0.91)		0.92 (0.87-0.97)	
Receptor status				
ER- and PR-positive and ERBB2-negative	1 [Reference]	<.001	1 [Reference]	<.001
ER-, PR-, and ERBB2-negative	1.60 (1.55-1.65)		1.54 (1.49-1.59)	
ERBB2-positive	1.36 (1.31-1.4)		1.17 (1.13-1.20)	
Health care setting				
Community	1 [Reference]	<.001	1 [Reference]	<.0001
Academic or research program	0.80 (0.78-0.82)		0.86 (0.85-0.89)	
Integrated Cancer Network Program	0.93 (0.91-0.95)		0.95 (0.93-0.98)	
Insurance type				
Medicaid, Medicare, or other government	1 [Reference]	<.001	1 [Reference]	<.001
Private or managed care	0.72 (0.70-0.74)		0.86 (0.83-0.87)	
Uninsured	0.94 (0.87-1.02)		1.04 (0.95-1.14)	
Total Charlson-Deyo comorbidity index score				
0	1 [Reference]	<.001	1 [Reference]	<.001
1	1.59 (1.56-1.63)		1.49 (1.47-1.54)	
2	2.44 (2.35-2.53)		2.18 (2.1-2.27)	
≥3	3.53 (3.35-3.73)		3.11 (2.94-3.30)	
Neighborhood-level median income, $				
<35 000	1 [Reference]	<.001	1 [Reference]	<.001
>35 000	0.78 (0.77-0.8)		0.91 (0.89-0.93)	
Neighborhood-level population with no high school degree, %				
<14	1 [Reference]	<.001	1 [Reference]	<.001
≥14	1.34 (1.31-1.36)		1.18 (1.16-1.21)	
Setting				
Metropolitan areas or urban	1 [Reference]	.09	1 [Reference]	.004
Rural	1.06 (0.99-1.13)		0.91 (0.84-0.97)	
GCC				
No	1 [Reference]	<.001	1 [Reference]	<.001
Yes	0.42 (0.41-0.43)		0.81 (0.77-0.85)	

Abbreviations: ER, estrogen receptor; ERBB2, human epidermal growth factor receptor 2 (formerly HER2); GCC, guideline-concordant care (assessed as time-dependent covariate in the multivariate analysis); HR, hazard ratio; PR, progesterone receptor.

### TTI

The TTI analytic cohort included 25 174 non-Hispanic Black patients and 233 357 non-Hispanic White patients. Of these, rates of TTI among non-Hispanic White patients were 46.9% within 30 days, 85.1% with 60 days, and 93.5% within 90 days, compared with 35.1% within 30 days, 74.6% within 60 days, and 88.6% within 90 days for non-Hispanic Black patients (eTable 2 in Supplement 1). In bivariate analysis, non-Hispanic White race, compared with non-Hispanic Black race, was associated with increased odds of initiating curative surgery or neoadjuvant within 30 days (OR, 1.65; 95% CI, 1.60-1.69), 60 days (OR, 2.11; 95% CI, 2.04-2.18), and 90 days (OR, 2.39;, 95% CI, 2.27-2.51) from date of breast cancer diagnosis.

## Discussion

In this cohort study using NCDB data on non-Hispanic Black and non-Hispanic White older adults with stage I to III breast cancer, we found that non-Hispanic Black race was associated with higher odds of nonreceipt of GCC after adjusting for demographics, tumor characteristics, comorbid illness, and individual and neighborhood levels of social determinants of health (SDOH). Non-Hispanic Black race was also associated with a greater risk for all-cause mortality. Importantly, our analysis also found that in our full model, racial disparities in all-cause mortality were reduced by adjusting for age, comorbid illness, tumor characteristics, insurance status, neighborhood-level SDOH, and receipt of GCC. Consistent with published literature, there were direct associations of age, comorbid illness, tumor characteristics, insurance status, and SDOH with all-cause mortality that were not mediated through GCC and not influenced by the association with race. The direct association of GCC with all-cause mortality at all levels of the exposure and covariates suggests that this is an important determinant of survival in older adults and further research is needed to evaluate these findings with a complete list of potential confounders examining frailty and functional status.

This study also highlights that most patients received GCC, which is likely reflective of predominance of Medicare coverage in this population of patients aged 65 years or older with breast cancer. This likely attenuates the effect of insurance coverage as a differential barrier to health access. However, it still demonstrates racial disparities in GCC, which have been established in the literature across multiple cancers.^26,27^ The association of GCC with all-cause mortality and potentially its role as a mediator in racial disparities in this analytic cohort of older adults emphasizes the need for nonbiased guideline adherence. Similar analysis of GCC using Surveillance, Epidemiology, and End Results Program claims data demonstrated that Black race was associated with a lower likelihood of receipt of guideline-concordant cancer curative treatment.^28^ Importantly, our study also found that the direct association of non-Hispanic Black race with all-cause mortality was reduced after adjusting for GCC and other covariates in the full model. Similar findings have been shown in analysis of disease-specific cancer mortality, which was not assessed in our study.^29^ These results are also supported by analysis of cohorts from the Veterans Health Administration health system that found no disparities in GCC in patients with colorectal cancer.^30^

These findings support strategies to optimize equitable receipt of GCC within health systems as a potentially modifiable mediator of breast cancer survival disparities.^31^ In older adults who are at a higher risk for nonreceipt of GCC, it is important to study the integration of geriatric assessments into decision-making for receipt of GCC.^32^ Although our analysis does not capture specific systemic therapy agents, prior studies with more granular data, such as relative dose intensity, have shown that older adults are also less likely to have optimal dose intensity therapy, which is associated with worse survival outcomes after adjusting for competing risks.^33^ Importantly, timeliness of therapy should also be addressed as a quality measure, which has been well established to be associated with worse survival outcomes.^34^

Other studies have shown that GCC does not attenuate these racial disparities in patients with breast cancer, eg, in a study of patients with stage I to III breast cancer in the Atlanta, Georgia, metropolitan area, receipt of GCC did not attenuate the mortality risk.^35^ Similar results were shown in some clinical trials.^36^ These results suggest that variables associated with racial disparities are likely multifactorial. Recent studies quantitatively assessing neighborhood deprivation indices, such as a 2023 study by Goel et al,^37^ have reported a strong association with cancer-specific survival after adjusting for GCC. Our adjusted model also included important neighborhood-level SDOH variables that were significantly associated with all-cause mortality. These findings emphasize recognition of neighborhood-level SDOH and their associations with cancer survival.^38^ It also underscores the importance of identifying potentially high-risk populations from disadvantaged neighborhoods where interventions may be important in mitigating unmet social needs and barriers to cancer care.^39^

Known clinical prognostic factors were associated with all-cause mortality eg, higher risk of mortality with triple-negative breast cancer and advanced stage disease.^2,19^ It is worth noting that ERBB2-positive disease was associated with lack of receipt of GCC but may be influenced by the lack of coded data for receipt of ERBB2-targeted therapy in the NCDB. Although year of diagnosis was significantly associated with both GCC and all-cause mortality, the associations were marginal and varied by year, and the clinical significance without more granular treatment and genomic diagnostic data was difficult to interpret.

### Limitations

This study has some limitations. First, the NCDB only includes information from Commission on Cancer–accredited hospitals, which may systematically underrepresent certain populations.^23^ Additionally, the data on systemic therapy lack granularity on systemic therapies and the use of novel diagnostic tests in clinical practice. Approximately 13% of patients had missing data on median income (12.9%), and high school completion rates (13.0%), but the proportion of missingness was equal in non-Hispanic Black and non-Hispanic White populations; therefore, missing variables were dropped during the regression analysis. Death was not considered as a competing risk in this analysis, which may bias our results. Additionally, by excluding patients with incomplete treatment data, we potentially excluded patients who died prior to treatment. However, this population comprised 2.8% of the cohort and likely reduces the magnitude of the bias. Furthermore, the NCDB does not include cancer-specific mortality; therefore, we were unable to ascertain breast cancer–specific mortality.^32^ Additionally, although we attempt to address potential mediators in our model, there are likely incompletely captured covariates included in our analysis, eg, our model includes Charles Deyo comorbidity index as a potential mediator; however, it does not completely capture frailty and performance status that influence cancer-directed treatment decisions in this population. Furthermore, there is incomplete capture of all components of neighborhood-level SDOH, and future studies examining validated composite measures of social vulnerability indices will need to be assessed as primary exposure variables in this population.

Despite these limitations, our analysis had several strengths. It was a large analysis of racial disparities in older adults who are at the intersection of disparities in receipt of GCC. We were able to adjust for clinical and demographic covariates, including neighborhood-level SDOH, which, in addition to GCC in our adjusted model, decreased the association of non-Hispanic Black race with all-cause mortality. This analysis offers support for the promotion of equitable GCC as a modifiable pathway for partly addressing some of the racial disparities among older adults with breast cancer.

## Conclusions

In this cohort study of older adults with stage I to III breast cancer, non-Hispanic Black race was associated with increased odds of not receiving GCC compared with non-Hispanic White race. In the adjusted Cox regression analysis, adjusting for the receipt of GCC, neighborhood-level SDOH and clinical and demographic variables reduced the direct association of race with all-cause mortality. Lastly, non-Hispanic White patients were more likely than non-Hispanic Black patients to initiate timely curative-intent treatment. Optimizing timely receipt of GCC may improve inferior survival outcomes among non-Hispanic Black older adults with breast cancer.

## Notes

Supplement 1. **eFigure 1.** Conceptual Diagram Illustrating Exposures, Outcomes and Covariates to Guide Model Interpretation **eTable 1.** Demographic and Clinical Characteristics of Patients Excluded Because of Missing Treatment Data **eFigure 2.** Kaplan-Meier Survival Analysis and Respective Life Tables of Non-Hispanic Black and Non-Hispanic White Patients, Aged 65 Years or Older With Stage I-III Breast Cancer **eTable 2.** Analysis of the Proportions of Patients Initiating Treatment Within 30, 60, and 90 Days From Diagnosis, Grouped by Race

Supplement 2.
Data Sharing Statement
