# Supplementary material for: Racial Disparities in Receipt of Guideline-Concordant Care in Older Adults With Early Breast Cancer
Source: JAMA Netw Open. 2024 Oct 24;7(10):e2441056. doi: 10.1001/jamanetworkopen.2024.41056 (PMC11581576; doi:10.1001/jamanetworkopen.2024.41056)
Supplement: Supplement 1. — eFigure 1. Conceptual Diagram Illustrating Exposures, Outcomes and Covariates to Guide Model Interpretation eTable 1. Demographic and Clinical Characteristics of Patients Excluded Because of Missing Treatment Data eFigure 2. Kaplan-Meier Survival Analysis and Respective Life Tables of Non-Hispanic Black and Non-Hispanic White Patients, Aged 65 Years or Older With Stage I-III Breast Cancer eTable 2. Analysis of the Proportions of Patients Initiating Treatment Within 30, 60, and 90 Days From Diagnosis, Grouped by Race [file jamanetwopen-e2441056-s001.pdf]

Supplemental Online Content

Castillo BS, Boadi T, Han X, Shulman LN, Martei YM. Racial disparities in receipt of guideline-concordant care in older adults with early breast cancer. *JAMA Netw Open*. 2024;7(10):e2441056. doi:10.1001/jamanetworkopen.2024.41056

eFigure 1. Conceptual Diagram Illustrating Exposures, Outcomes and Covariates to Guide Model Interpretation

eTable 1. Demographic and Clinical Characteristics of Patients Excluded Because of Missing Treatment Data

eFigure 2. Kaplan-Meier Survival Analysis and Respective Life Tables of Non-Hispanic Black and Non-Hispanic White Patients, Aged 65 Years or Older With Stage I-III Breast Cancer

eTable 2. Analysis of the Proportions of Patients Initiating Treatment Within 30, 60, and 90 Days From Diagnosis, Grouped by Race

This supplemental material has been provided by the authors to give readers additional information about their work.

© 2024 Castillo BS et al. *JAMA Network Open.*

eFigure 1 Conceptual diagram illustrating exposures, outcomes and covariates to guide model interpretation

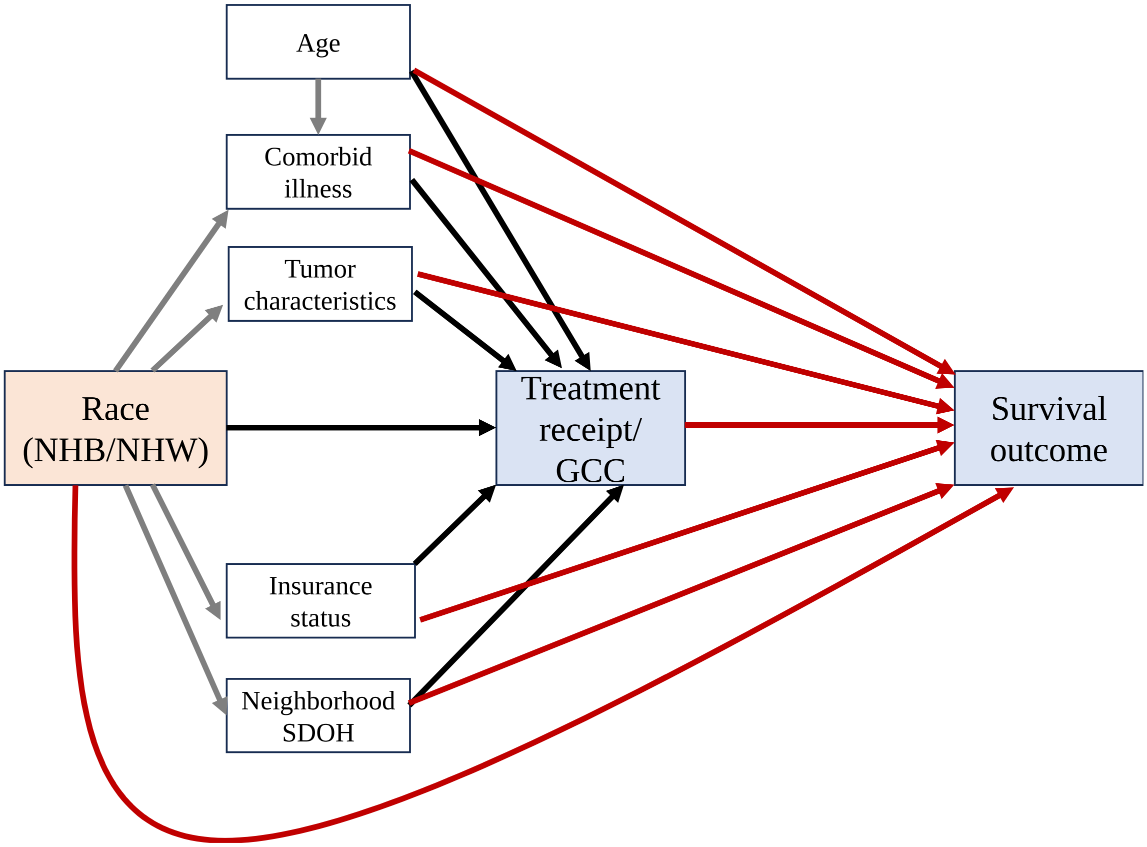

Key: GCC guideline concordant care, SDOH neighborhood-level social determinants of health, NHB non-Hispanic Black, NHW non-Hispanic White.
We considered tumor characteristics (stage and receptor status), comorbid illness (Charlson-Deyo), insurance status, neighborhood level social determinants of health (educational attainment and median income) and GCC as mediators of the association between race on survival outcomes. In our model race was the primary exposure. Variables not measured in the study were frailty, performance status, genetics, and a more comprehensive assessment of individual socioeconomic status and neighborhood SDOH. Red arrows represent associations with survival outcomes, black arrows represent associations with GCC and grey arrows represent associations between race and covariates included in our analysis.

© 2024 Castillo BS et al. *JAMA Network Open.*

eTable 1 Demographic and clinical characteristics of patients excluded because of missing treatment data

		All Groups(N= 7555)	White(N=6639)	Black(N= 916)
Age>=75	0:65<=age<75	5063 (67.0%)	4423 (66.6%)	640 (69.9%)
	1:age>=75	2492 (33.0%)	2216 (33.4%)	276 (30.1%)
Disease Stage	1:stage I	4639 (61.4%)	4168 (62.8%)	471 (51.4%)
	2:stage II	2368 (31.3%)	2000 (30.1%)	368 (40.2%)
	3:stage III	548 (7.3%)	471 (7.1%)	77 (8.4%)
Age (Years)	Max	90.0	90.0	90.0
	Mean	72.5	72.6	72.0
	Median	71.0	72.0	71.0
	Min	65.0	65.0	65.0
	N	7555	6639	916
	Q1	68.0	68.0	67.0
	Q3	76.0	76.0	76.0
	STD	5.9	5.9	5.7
ER Status	1:positive	6157 (81.5%)	5515 (83.1%)	642 (70.1%)
	2:negative	1319 (17.5%)	1060 (16.0%)	259 (28.3%)
	3:Unknown/missing	79 (1.0%)	64 (1.0%)	15 (1.6%)
PR Status	1:positive	5343 (70.7%)	4804 (72.4%)	539 (58.8%)
	2:negative	2125 (28.1%)	1764 (26.6%)	361 (39.4%)
	3:Unknown/missing	87 (1.2%)	71 (1.1%)	16 (1.7%)
HER2 Status	1:positive	1030 (13.6%)	891 (13.4%)	139 (15.2%)
	2:negative	6400 (84.7%)	5648 (85.1%)	752 (82.1%)
	3:Unknown/missing	125 (1.7%)	100 (1.5%)	25 (2.7%)
Time(months) from diagnosis to death/follow-up	Max	135.8	135.8	134.3
	Mean	61.5	62.2	56.5
	Median	57.5	58.5	51.5
	Min	0.0	1.8	0.0
	N	7555	6639	916
	Q1	38.2	38.7	34.6
	Q3	84.6	85.6	76.4
	STD	31.1	31.1	30.6
Year of Diagnosis	2010	830 (11.0%)	734 (11.1%)	96 (10.5%)
	2011	909 (12.0%)	818 (12.3%)	91 (9.9%)
	2012	862 (11.4%)	765 (11.5%)	97 (10.6%)

© 2024 Castillo BS et al. *JAMA Network Open.*

		All Groups(N= 7555)	White(N=6639)	Black(N= 916)
	2013	846 (11.2%)	753 (11.3%)	93 (10.2%)
	2014	874 (11.6%)	775 (11.7%)	99 (10.8%)
	2015	833 (11.0%)	720 (10.8%)	113 (12.3%)
	2016	1007 (13.3%)	876 (13.2%)	131 (14.3%)
	2017	1394 (18.5%)	1198 (18.0%)	196 (21.4%)
Insurance Payer	0:Not Insured	30 (0.4%)	21 (0.3%)	9 (1.0%)
	1:Private Insurance/Managed Care	1007 (13.3%)	852 (12.8%)	155 (16.9%)
	2:Medicaid	108 (1.4%)	70 (1.1%)	38 (4.1%)
	3:Medicare	6202 (82.1%)	5508 (83.0%)	694 (75.8%)
	4:Other Government	28 (0.4%)	27 (0.4%)	1 (0.1%)
	9:Insurance Status Unknown	180 (2.4%)	161 (2.4%)	19 (2.1%)
Percent no high school degree	.	839 (11.1%)	713 (10.7%)	126 (13.8%)
	0:<14%	2508 (33.2%)	2378 (35.8%)	130 (14.2%)
	1:>=14%	4208 (55.7%)	3548 (53.4%)	660 (72.1%)
Median Income Quartiles	.	821 (10.9%)	702 (10.6%)	119 (13.0%)
	0:<$35,000	1983 (26.2%)	1601 (24.1%)	382 (41.7%)
	1:>=$35,000	4751 (62.9%)	4336 (65.3%)	415 (45.3%)
FACILITY TYPE	1:Community	4222 (55.9%)	3846 (57.9%)	376 (41.0%)
	2:Academic/Research Program	1920 (25.4%)	1543 (23.2%)	377 (41.2%)
	3:Integrated Network Cancer Program	1413 (18.7%)	1250 (18.8%)	163 (17.8%)
Urban/Rural	.	331 (4.4%)	306 (4.6%)	25 (2.7%)
	1:Metro areas/urban	7074 (93.6%)	6196 (93.3%)	878 (95.9%)
	2:Completely rural	150 (2.0%)	137 (2.1%)	13 (1.4%)
Charlson-Deyo Score	0:Total Charlson-Deyo Score of 0	5910 (78.2%)	5265 (79.3%)	645 (70.4%)
	1:Total Charlson-Deyo Score of 1	1239 (16.4%)	1044 (15.7%)	195 (21.3%)
	2:Total Charlson-Deyo Score of 2	279 (3.7%)	229 (3.4%)	50 (5.5%)
	3:Total Charlson-Deyo Score of 3 or more	127 (1.7%)	101 (1.5%)	26 (2.8%)
Medicaid Expansion status	0:Non-Expansion States	3102 (41.1%)	2704 (40.7%)	398 (43.4%)
	1:January 2014 Expansion States	2132 (28.2%)	1859 (28.0%)	273 (29.8%)
	2:Early Expansion States(2010-2013)	1150 (15.2%)	1028 (15.5%)	122 (13.3%)
	3:Late Expansion States(after Jan. 2014)	1171 (15.5%)	1048 (15.8%)	123 (13.4%)
Days from Diagnosis to Surgery	Max	372.0	372.0	308.0
	Mean	34.6	33.9	40.6
	Median	30.0	29.0	34.0

© 2024 Castillo BS et al. *JAMA Network Open.*

		All Groups(N= 7555)	White(N=6639)	Black(N= 916)
	Min	0.0	0.0	0.0
	N	6422	5688	734
	Q1	20.0	20.0	20.0
	Q3	44.0	43.0	51.0
	STD	27.8	26.5	36.3
Days from Diagnosis to Chemo	Max	504.0	504.0	476.0
	Mean	49.2	46.7	60.9
	Median	35.0	34.0	43.0
	Min	0.0	0.0	3.0
	N	811	670	141
	Q1	25.0	24.0	30.0
	Q3	51.0	48.0	63.0
	STD	54.8	53.1	61.2
Receptor_status		131 (1.7%)	105 (1.6%)	26 (2.8%)
	1:(ER_positive OR PR_positive) AND HER2_	5527 (73.2%)	4954 (74.6%)	573 (62.6%)
	2:HER2_positive	1030 (13.6%)	891 (13.4%)	139 (15.2%)
	3:ER_negative AND PR_negative AND HER2_n	867 (11.5%)	689 (10.4%)	178 (19.4%)
Vital status	0:dead	1683 (22.3%)	1459 (22.0%)	224 (24.5%)
	1:alive	5872 (77.7%)	5180 (78.0%)	692 (75.5%)

© 2024 Castillo BS et al. *JAMA Network Open.*

eFigure 2: Kaplan Meier survival analysis and respective life tables of non-Hispanic Black and non-Hispanic White patients, ≥65 years old with stage I-III breast cancer patients

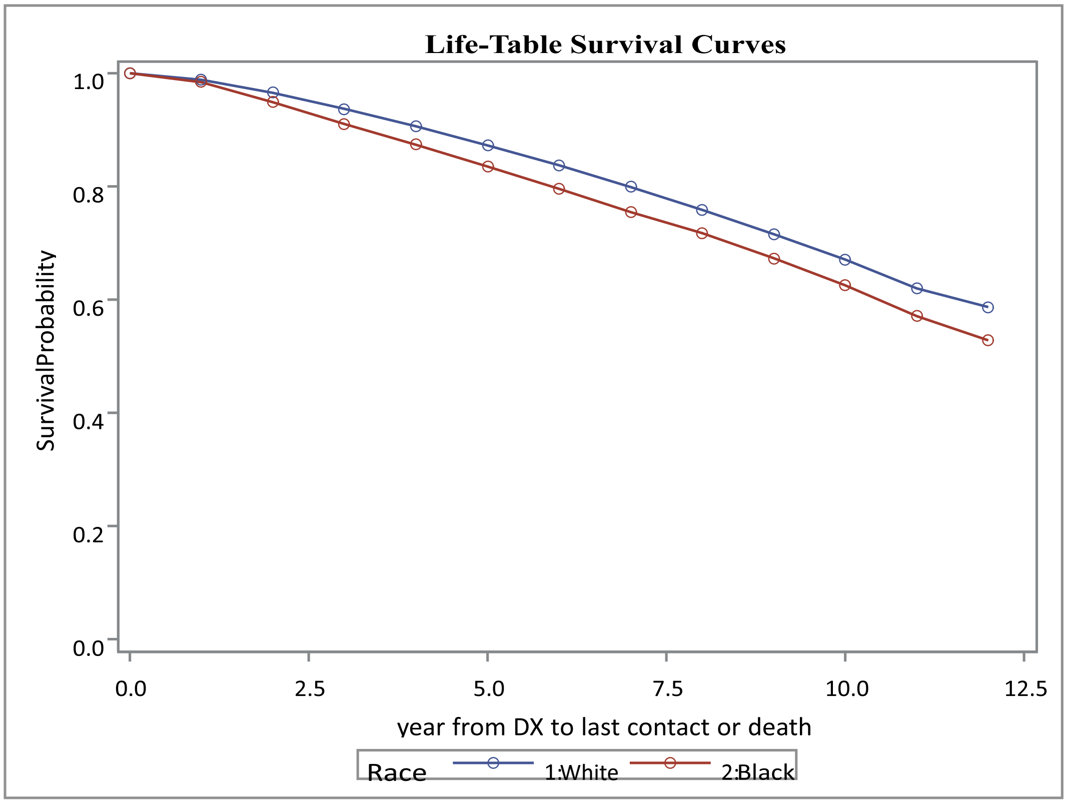

© 2024 Castillo BS et al. *JAMA Network Open.*

eFigure 2: Kaplan Meier survival analysis and respective life tables of non-Hispanic Black and non-Hispanic White patients, ≥65 years old with stage I-III breast cancer patients (continued)

Life Table Survival Estimates<br>Stratum 1: Race = 1:White									
Interval									
[Lower,	Upper)	Number Failed	Number Censored	Effective Sample Size	Conditional Probability of Failure	Conditional Probability Standard Error	Survival	Failure	Survival Standard Error
0	1	2694	2176	232265.0	0.0116	0.000222	1.0000	0	0
1	2	5302	3341	226812.5	0.0234	0.000317	0.9884	0.0116	0.000222
2	3	6277	11093	214293.5	0.0293	0.000364	0.9653	0.0347	0.000381
3	4	6155	30464	187238.0	0.0329	0.000412	0.9370	0.0630	0.000511
4	5	5595	31751	149975.5	0.0373	0.000489	0.9062	0.0938	0.000627
5	6	4587	28993	114008.5	0.0402	0.000582	0.8724	0.1276	0.000749
6	7	3795	24566	82642.0	0.0459	0.000728	0.8373	0.1627	0.000880
7	8	2820	21330	55899.0	0.0504	0.000926	0.7989	0.2011	0.00104
8	9	1910	17273	33777.5	0.0565	0.00126	0.7586	0.2414	0.00123
9	10	1033	13550	16456.0	0.0628	0.00189	0.7157	0.2843	0.00150
10	11	369	7584	4856.0	0.0760	0.00380	0.6707	0.3293	0.00195
11	12	19	676	357.0	0.0532	0.0119	0.6198	0.3802	0.00312
12	.	0	0	0.0	0	0	0.5868	0.4132	0.00794

© 2024 Castillo BS et al. *JAMA Network Open.*

eFigure 2: Kaplan Meier survival analysis and respective life tables of non-Hispanic Black and non-Hispanic White patients, ≥65 years old with stage I-III breast cancer patients (continued)

Life Table Survival Estimates<br>Stratum 2: Race = 2:Black									
Interval									
[Lower,	Upper)	Number Failed	Number Censored	Effective Sample Size	Conditional Probability of Failure	Conditional Probability Standard Error	Survival	Failure	Survival Standard Error
**0**	**1**	399	238	25055.0	0.0159	0.000791	1.0000	0	0
**1**	**2**	875	406	24334.0	0.0360	0.00119	0.9841	0.0159	0.000791
**2**	**3**	917	1259	22626.5	0.0405	0.00131	0.9487	0.0513	0.00140
**3**	**4**	782	3373	19393.5	0.0403	0.00141	0.9102	0.0898	0.00183
**4**	**5**	670	3403	15223.5	0.0440	0.00166	0.8735	0.1265	0.00218
**5**	**6**	538	2913	11395.5	0.0472	0.00199	0.8351	0.1649	0.00254
**6**	**7**	420	2509	8146.5	0.0516	0.00245	0.7957	0.2043	0.00293
**7**	**8**	267	2099	5422.5	0.0492	0.00294	0.7546	0.2454	0.00340
**8**	**9**	200	1731	3240.5	0.0617	0.00423	0.7175	0.2825	0.00392
**9**	**10**	110	1263	1543.5	0.0713	0.00655	0.6732	0.3268	0.00477
**10**	**11**	40	685	459.5	0.0871	0.0132	0.6252	0.3748	0.00625
**11**	**12**	3	74	40.0	0.0750	0.0416	0.5708	0.4292	0.0100
**12**	**.**	0	0	0.0	0	0	0.5280	0.4720	0.0255

© 2024 Castillo BS et al. *JAMA Network Open.*

eTable 2: Analysis of the proportions of patients initiating treatment within 30, 60 and 90 days from diagnosis, grouped by race

Time to treatment initiation target	Non-Hispanic White (n=233357)	%	Non-Hispanic Black (25174)	%	OR (95% CI)	p value
Treatment initiation within 30 days	109623	46.9	8844	35.1	1.65 (1.6-1.69)	p<0.001
Treatment initiation within 60 days	198561	85.1	18789	74.6	2.11 (2.04-2.18)	p<0.001
Treatment initiation within 90 days	218089	93.5	22309	88.6	2.39 (2.27-2.51)	p<0.001

© 2024 Castillo BS et al. *JAMA Network Open.*
