# Supplementary material for: Racial Disparities in Receipt of Guideline-Concordant Care in Older Adults With Early Breast Cancer
Source: JAMA Netw Open. 2024 Oct 24;7(10):e2441056. doi: 10.1001/jamanetworkopen.2024.41056 (PMC11581576; doi:10.1001/jamanetworkopen.2024.41056)
Supplement: Supplement 2. — Data Sharing Statement [file jamanetwopen-e2441056-s002.pdf]

Data Sharing Statement

Data

Data available: No

Additional Information

Explanation for why data not available: The cohort analysis utilizes publicly available NCDB data.
